# Design and Experimental Investigation of a Rotational Piezoelectric Energy Harvester with an Offset Distance from the Rotation Center

**DOI:** 10.3390/mi13030388

**Published:** 2022-02-28

**Authors:** Jun Chen, Xiangfu Liu, Hengyang Wang, Sheng Wang, Mingjie Guan

**Affiliations:** Department of Instrumental and Electrical Engineering, Xiamen University, Xiamen 361005, China; 35120200156025@stu.xmu.edu.cn (J.C.); liuxiangfu@stu.xmu.edu.cn (X.L.); 35020201151416@stu.xmu.edu.cn (H.W.); shengwong@stu.xmu.edu.cn (S.W.)

**Keywords:** piezoelectric, energy harvesting, rotational, offset distance, cantilever beam

## Abstract

Rotational energy harvesting technology has attracted more and more attention recently. This paper presents a piezoelectric rotational energy harvester that can be mounted with an offset distance from the rotation center. The piezoelectric energy harvester is designed to be dynamically excited by the force due to gravity, which causes the piezoelectric cantilever beams in the harvester to vibrate periodically as the harvester rotates. A novel design of the harvester structure with a hollow mass is proposed and analyzed in this paper. Experiments were performed to investigate the design and analysis. A power output of 106~2308 μW can be achieved at the rotating frequencies of 0.79~14 Hz with a piezoelectric cantilever beam in the prototyped energy harvester. Results showed that the prototyped harvester can be mounted on a rotating wheel hub and output sufficient power in a wide frequency range for wireless monitoring sensors.

## 1. Introduction

Technologies of kinetic energy harvesting have achieved rapid progress in recent decades due to their ability to harvest ambient kinetic energy to generate electricity. Among the kinetic motion, rotational motion has been widely used for various applications, including engines [1], turbines [2,3,4,5], rotating shafts [6], human motions [7,8,9,10] and vehicle wheels [11,12,13]. Fu et al. [14] reviewed the state-of-the-art technology in the field of rotational energy harvesting for self-powered sensing. However, studies on rotational energy harvesting are still not to the same extent as those of vibration energy harvesting. Due to the centrifugal force in rotational environments, many vibration-based energy harvesting methods are not applicable. Among various kinetic energy harvesting mechanisms, the piezoelectric energy harvester has attracted the most attention due to its relatively high power density, compact structure and scalability from micro to human scale.

At present, research on rotational piezoelectric energy harvesting mainly includes methods based on mechanical plucking, magnetic excitation, and gravity. In the mechanical plucking method, strikers are usually used to pluck the cantilever beams to excite them [15,16]. However, with the mechanical plucking mechanism, the contact parts of the mechanical structures will easily wear out after long-time use, which will cause the harvester to easily fail [16]. Similar to the mechanical plucking method, the magnetic excitation method utilizes the magnetic force generated by the magnets close to the piezoelectric structures to generate excitation [17,18,19,20]. However, the magnetic excitation method is unsuitable for some magnetic-sensitive applications, and the magnetic device is usually bulky. The gravity method uses the gravity of the mass to motivate vibration. This method does not need the additional auxiliary structures for plucking, and the whole structure can be more compact. Our research is based on the gravity method. The gravity method has two basic layouts. One layout is that the clamp of the beam is closer to the rotation center than the mass, which is also called an outward layout. Rui et al. [21] utilized such a layout for energy harvesting in a wheel spike. The beam stiffness increases due to the centrifugal force of the rotational mass in this manner. The resonance frequency of the beam increases while the rotation frequency increases, such that a self-tuning mechanism is achieved. The other layout is that the mass of the beam is closer to the rotation center than the clamped end of the beam, which is also called an inward layout. Mei et al. [22] applied such a layout in their research. The stiffness of the beam can be decreased by the centrifugal force of the mass in this manner, which is called the centrifugal softening effect. Hsieh et al. [23] investigated various cantilever configurations for rotational piezoelectric energy harvester. They found that centrifugal force plays an important role for passive self-tuning of resonance. An in-depth comparison of the centrifugal softening and stiffening effects of the rotational energy harvesters is presented by Fang et al. [24] with a dimensionless analytical model. In whichever layout is used, the limitation of vibration amplitude of the beam is needed, otherwise the vibration amplitude of the piezoelectric beam will exceed the limit of the piezoelectric materials. Our previous work [25] is similar to the inward layout and made the mass center coincide with the rotation center to achieve a high vibration amplitude. It had been proven to be workable, but there was the problem that the mass should be mounted close to the center of the rotation. However, in some applications, the neighborhood of the rotation center was occupied by the rotation hub or shaft, and the energy harvester including the mass should be mounted a distance offset from the rotation center. Under the circumstances, the centrifugal force will become much larger and make a feasible design of the harvester more complicated. Wang et al. [26] proposed a method of using magnets to improve the performance of a high-efficiency compressive-mode piezoelectric energy harvester with the offset configuration in rotational environments. However, additional auxiliary structures are needed to generate the magnetic field. This paper is aimed at introducing an innovative design of rotational piezoelectric energy harvester that can be mounted at a distance offset from the rotational center without using the magnetic device. It can generate a high output voltage and high output power in a wide rotation frequency range.

The organization of this paper is as follows. In Section 2, the system design and theoretical analysis of the rotational piezoelectric energy harvester are presented. In Section 3, a prototype of the rotational piezoelectric energy harvester is built, and experiments are carried out to demonstrate the performances of the energy harvester mounted on a rotational wheel hub. The conclusions and discussions are given in Section 4.

## 2. System Design and Theoretical Analysis

**Figure 1:**
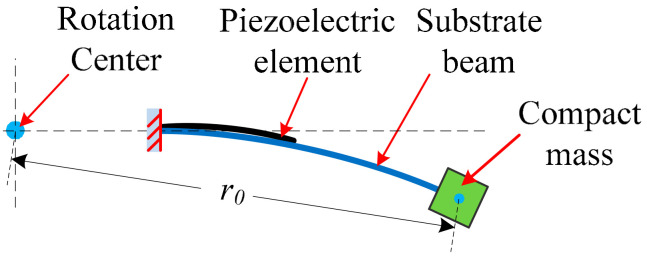
Schematic of a conventional cantilever beam structure with compact mass in a rotational energy harvester.

In our design, similar to our previous design [25], a cantilever beam structure is used to increase the strain delivered to a piezoelectric element on the surface of the cantilever, and a metal beam is used as the substrate beam to achieve a high robustness. Based on the cantilever beam structure, a conventional rotational piezoelectric energy harvester with a compact mass is shown in Figure 1 [11,21].

From Figure 1, it can be seen that when the object rotates at a constant speed ω_0_, the centrifugal force *F_cent_* of the rotating object is given by

*F_cent_* = *m*_0_*ω*_0_^2^*r*_0_, (1)

where *m*_0_ is the mass of the rotating object, and *r*_0_ is the radius of the rotation. The centrifugal force is oriented away from the axis of the rotation.

When the rotation axis is parallel to the Earth’s surface, the rotational beam and mass will be subjected to an excitation from the Earth, which is around ±1 g. For a conventional rotational energy harvester, when the energy harvester rotates in a common frequency range (e.g., 5–10 Hz for a vehicle wheel), the centrifugal force of the rotational mass will be much larger than its gravitational force. A centrifugal stiffening effect will exist, and only small transverse vibrations of the beam can be generated.

**Figure 2:**
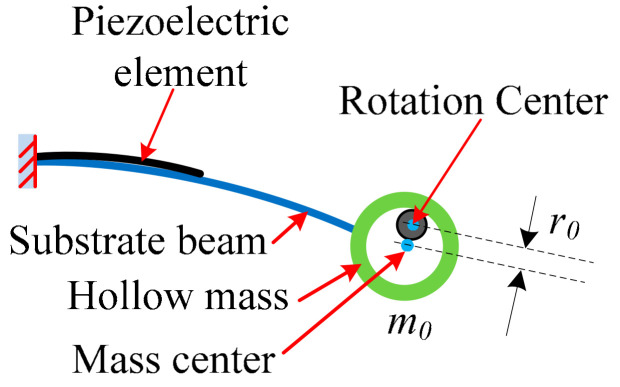
Schematic of the designed cantilever beam with hollow mass.

In order to increase the beam vibration amplitude, our previous design [25] was to reduce the amplitude of centrifugal force by greatly decreasing the radius of the rotation. However, in some rotation applications, the compact mass should be mounted a distance offset from the rotational center. The schematic of our new design is shown in Figure 2. In principle, the compact mass is replaced by a hollow mass, and the center of the hollow mass coincides with the rotation center if there is no gravity. The rotational hub becomes a constrained frame for the hollow mass to constrain the vibration amplitude of the beam. Without gravity, the gaps between the rotational hub and hollow mass are symmetrically distributed. With this design, the rotation radius *r*_0_ will be greatly decreased. Therefore, the centrifugal force of the rotational mass will be greatly decreased too.

**Figure 3:**
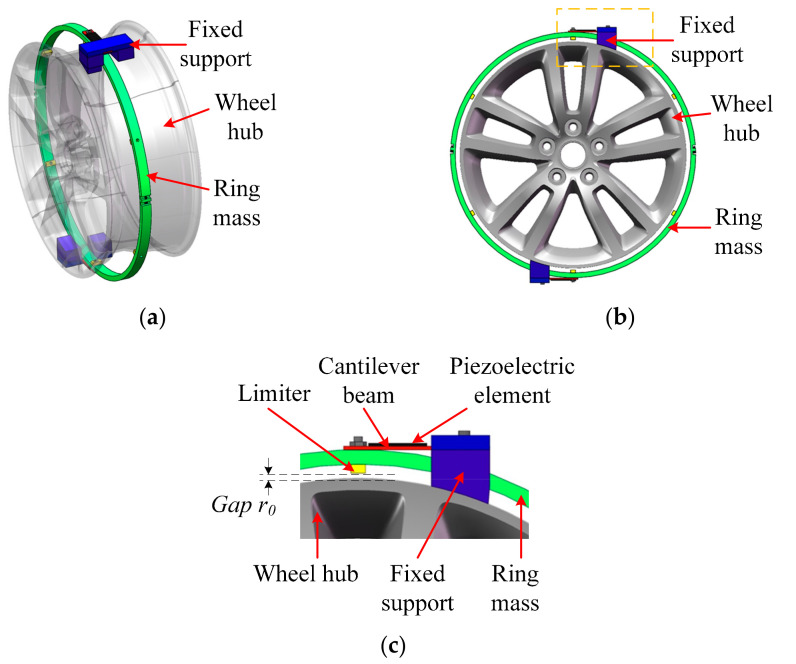
Structure of wheel and the rotational energy harvester: (**a**) three-dimensional view; (**b**) front view; (**c**) close view.

Based on this idea, a design of the rotational energy harvester comprising two piezoelectric cantilever beams and one hollow mass mounted on the wheel hub is shown in Figure 3. The beam orientation is adjusted according to the rotation structure. As the wheel-mounted energy harvester is used mainly to support power for the tire pressure sensors, the energy harvester should be ideally mounted outside the wheel hub and inside the tire. In Figure 3a, a three-dimensional view of the harvester and the wheel hub is shown. Usually, there is a groove on the wheel hub, and the designed hollow mass of the harvester can be mounted in the groove. The hollow mass is constructed by a ring-like mass, which can be separated into two parts during fabrication and mounted together as a ring during working. The inner radius of the ring mass is larger than the outer radius of the groove of the wheel hub where the ring is mounted. A front view of the structure is shown in Figure 3b, and a close view of the piezoelectric cantilever beam is shown in Figure 3c. As shown in Figure 3b, two cantilever beams are used to mount the mass, and the planes of the beams are parallel with the rotation axis but do not coincide with it. Two fixed supports are mounted on the wheel hub tightly. For both cantilever beams, one end is fixed on the support and the other end is fixed on the ring mass. Between the ring mass and wheel hub, there are six limiters mounted on the inside cylindrical surface of the ring. As shown in Figure 3c, the gap between the limiters and the hub can be adjusted by the height of the limiters. The gaps between the limiters and the hub should be equally distributed when there is no gravity.

**Figure 4:**
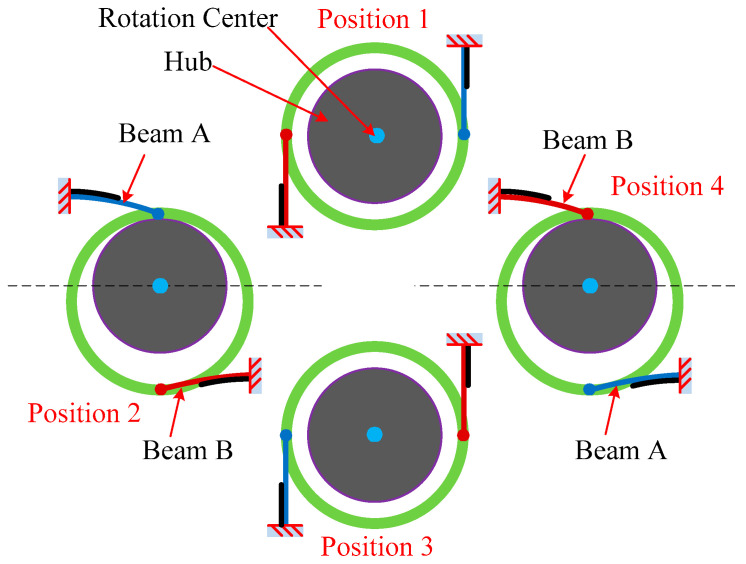
Schematic diagram showing four beam positions.

A schematic of the working principle of the energy harvester with four beam positions during rotation is shown in Figure 4. At “Position 1” and “Position 3”, two beams are both in the vertical direction. Therefore, both beams have no displacements in the horizontal direction. At “Position 2”, due to the gravity, the ring mass descends but is stopped by the wheel hub. The piezoelectric element of “Beam A” is stretched and the piezoelectric element of “Beam B” is compressed. On the contrary, at “Position 4”, the piezoelectric element of “Beam A” is compressed, and the piezoelectric element of “Beam B” is stretched. From Figure 4, it can be seen that the gravitational force on the hollow mass causes periodic vibrations of the beams when the beams are rotating.

**Figure 5:**
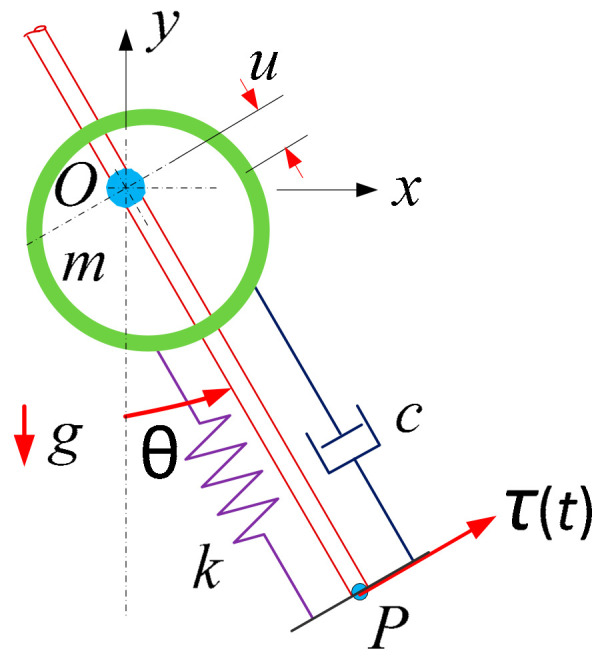
Schematic of the system in a structural dynamic analysis.

Similar to the analysis in our previous work [25], the structural dynamic analysis can be explained in a schematic as shown in Figure 5. Under the assumption that the energy harvester beams vibrate in small amplitudes and the hollow mass is much larger than the mass of the cantilever beams, the hollow mass can be approximately treated as an object moving back and forth linearly in a small range. The restoring forces of two bended cantilever beams acting on the hollow mass can be regarded as a spring force in the transverse direction. An imaginary massless rigid rod is pivoted about point *O*. The equivalent moves back and forth along the rod. An equivalent spring with stiffness *k* and an equivalent damper with damping coefficient *c* connect the mass *m* and the end point *P* of the rod. The spring will be at its original length when the mass *m* is at the point *O*. As shown in Figure 5, the spring is shortened by a length *u*. A torque *τ(t)* perpendicular to the rod is applied at the end point *P* to drive the rod.

Through Lagrange’s equations, the system equations of motion can be written as

(2) $$m\frac{d^{2}u}{dt^{2}}+c\frac{du}{dt}+(k-m\omega^{2})u=mg\cos(\omega t).$$

Equation (2) implies that under the small vibration amplitude, the vibrating structure can be regarded as a damped system under harmonic excitations. When the vibration amplitude reaches the gap distance, the limiters and the wheel hub will become the rigid stopper of the vibration. From Equation (2), the magnitude of the base acceleration is equal to *g*.

**Figure 6:**
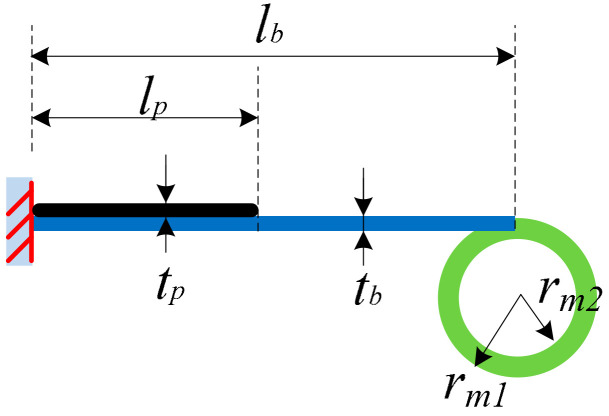
Dimensions of the beam structure and the ring mass (not in scale).

The dimensions of one cantilever beam and the ring mass (not in scale with the real structure) are shown in Figure 6. *l_b_*, *w_b_* and *t_b_* are the length, width and thickness of the substrate beam; *l_p_*, *w_p_* and *t_p_* are the length, width and thickness of the piezoelectric element; *r_m_*_1_ and *r_m_*_2_ are the outer radius and inner radius of the ring mass; the width of the ring mass is *w_m_*. In order to avoid that the beam and mass are stuck at some place due to an overlarge centrifugal force, the centrifugal force *F_cent_* of the rotating parts should be smaller than the gravity of the rotating parts. Therefore, we have a constraint on the radius of the rotation *r*_0_ that *r*_0_ < *g/*ω_0_^2^. Using a rotating frequency of 15 Hz as an example, the radius of the rotation should be smaller than 1.103 mm. In our design, the gap, i.e., the radius of the rotation *r*_0_ is designed at 1 mm considering the application. The rotational mass is not related to the radius of the rotation *r*_0_, but it will influence the vibration acceleration of the cantilever beam. A larger rotational mass will increase the vibration acceleration of the beams and therefore increase the voltage outputs of the piezoelectric elements. In our design, a large rotational mass is used. In the design, to guarantee the reliability of the structure, the mechanical strains of the piezoelectric elements and other structures should be kept within material specifications.

## 3. Experimental Validations

**Figure 7:**
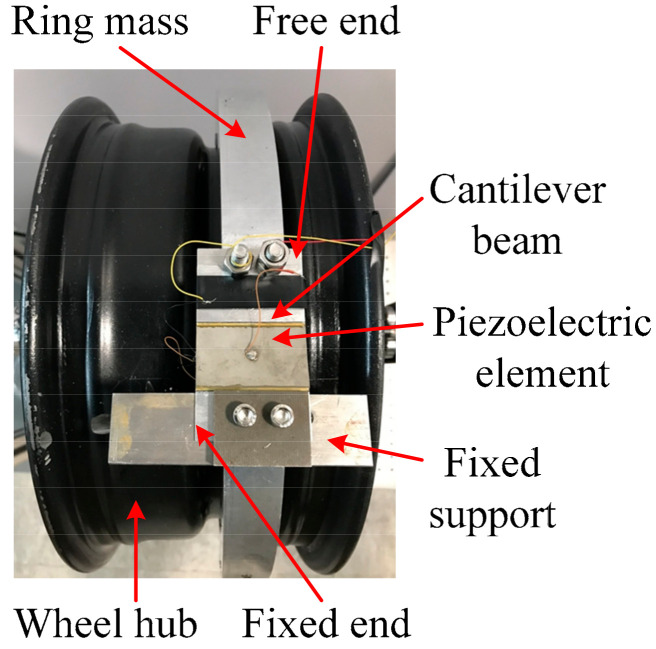
Prototyped rotational piezoelectric energy harvester mounted on a wheel hub.

A prototype of the designed rotational piezoelectric energy harvester mounted on a wheel hub is shown in Figure 7. The piezoelectric elements used in the prototype are piezoelectric ceramics. Both of the substrate beams and the ring mass are made of aluminum. As the piezoelectric ceramics are fragile, only the substrate beams are clamped by the fixed supports. Two piezoelectric elements, modeled PSI-5A4E, (Piezo Systems, Woburn, MA, America) are surface-bonded on the outer side of both beams by an enhanced coupling approach with edge elements [27]. There is a small distance *x*_0_ between the piezoelectric elements and the respective fixed supports. Dimensions of the beam structure and piezoelectric elements are given in Table 1, where *m_ring_* is the mass of the ring mass; *r_g_* is the external radius of the groove in the wheel hub; *h*_l_ is the height of the limiter. In Table 1, the inner radius of the ring mass *r_m_*_2_ is 170 mm, the external radius of the groove in the wheel hub *r_g_* is 159 mm, and the height of the limiter *h*_l_ is 10 mm. Therefore, the gap distance *r*_0_ is 1 mm.

**Figure 8:**
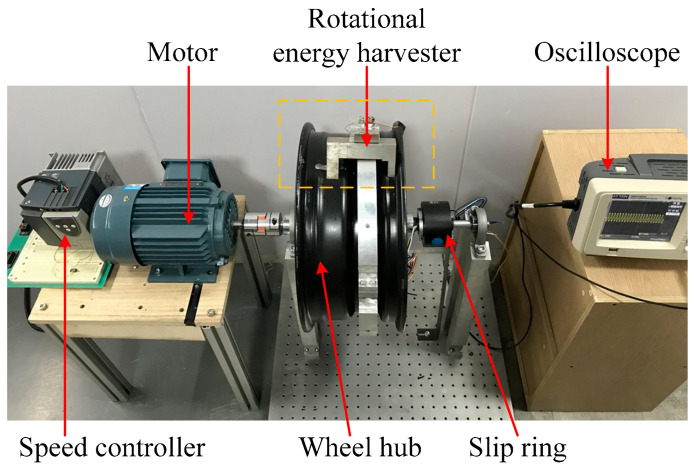
Experimental setup to test the energy harvester.

The experimental setup is shown in Figure 8. The prototyped rotational piezoelectric energy harvester is mounted on a wheel hub rotated by a three-phase AC motor, modeled YE3-80M-2-0.75, (Gexin Electrical, Nanjing, China). The rotation speed is controlled by a speed controller, modeled ATV11 (Schneider, Rueil, France). The output voltages of the piezoelectric elements are connected with the use of a slip ring.

**Figure 9:**
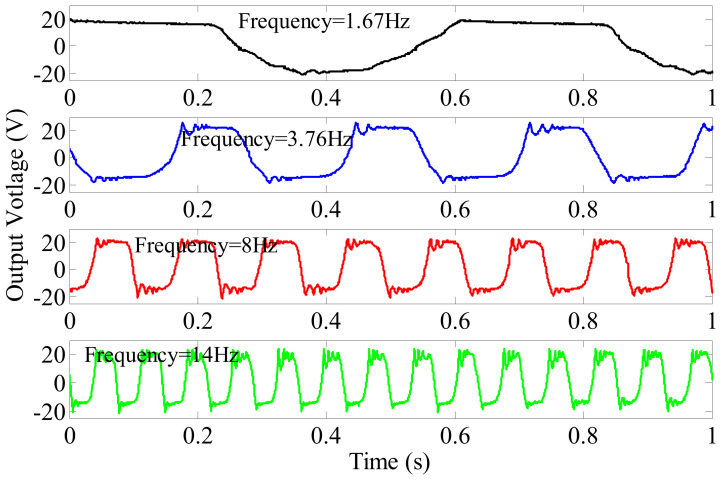
Piezoelectric voltage output versus time.

Using the piezoelectric element mounted on “Beam A”, for example, the output open-circuit voltage waves of the piezoelectric element at rotating frequencies ranging from 1.67 to 14 Hz are shown in Figure 9. From Figure 9, it can be found that the output open-circuit voltage waves are similar to a square wave in the shown cases, except for the one at the low frequency of 1.67 Hz. At low frequency, the negative half cycles are similar to a sinusoid curve; however, the positive half cycles are in saturation condition. This implies that the piezoelectric cantilever beam is closer to the stopper at one side than the other side under no gravity condition. If the piezoelectric cantilever beam is adjusted accordingly, the positive and negative half cycle of the output voltage at low frequency can be more symmetrical. The peak open-circuit voltages are almost the same as the rotating frequency increases, which is different from our previous work [25]. The reason may be that the ring mass in this prototype is much larger than the mass used in our previous work. Therefore, the vibration acceleration is larger and the ring mass will reach the stoppers more rapidly.

When the designed rotational energy harvester is mounted onto a rotating wheel hub, the vehicle speed will be related to the rotating frequency of the wheel hub and the tire model. In this research, it is assumed that tires modeled “195/65/15” are applied to the wheel, with diameters of 634.5 mm. From Figure 9, it is observed that the peak voltage is around 20 V at a rotating frequency as low as 1.67 Hz (corresponding to a vehicle speed of 11.98 km/h). Considering that the wireless transmitter usually used a 1.8~3 V power supply, the output voltage of the prototype at frequency 1.67 Hz is large enough for a rectifier circuit to charge the energy storage element.

**Figure 10:**
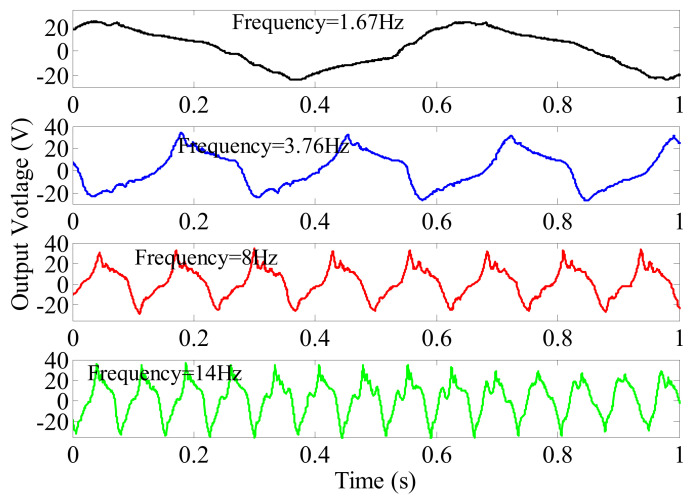
External voltages with resistors versus rotating frequency.

In our research, in order to measure the harvested power, external resistors are used to connect the piezoelectric elements in parallel in the energy harvesting circuit. The optimal resistive load *R_opt_* under a particular frequency *f_n_* is *R_opt_ =* 1/(*2πf_n_C_p_*), where *C_p_* is the capacitance of the piezoelectric element. The output voltages with respective external resistors at several rotating frequencies are shown in Figure 10. The external resistors used are 839.7, 372.9, 175.3 and 100.2 kΩ for the rotating frequencies 1.67, 3.76, 8 and 14 Hz, respectively. From Figure 10, it can be found that the waves of the output voltages with external resistors became steeper than the open-circuit voltage curves shown in Figure 9. The waves at the high frequencies of 8 and 14 Hz are similar to a sawtooth wave.

**Figure 11:**
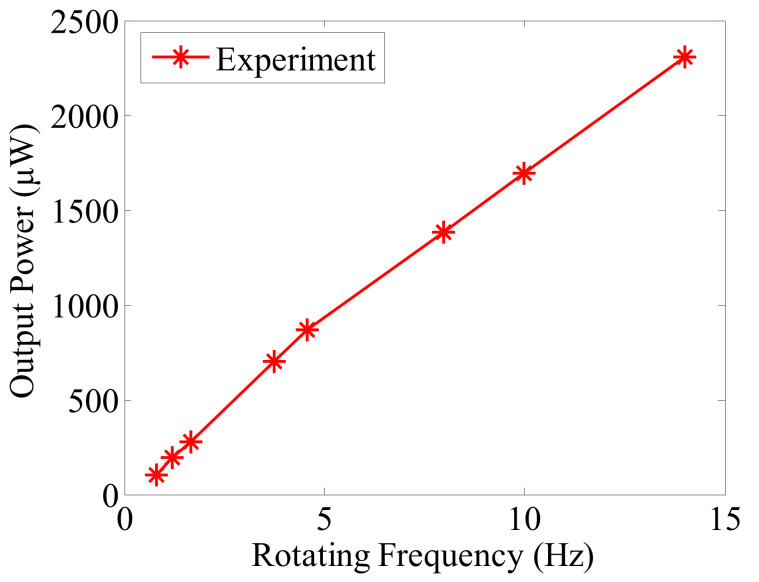
Piezoelectric power output versus rotating frequency.

The power outputs transferred to the external resistive loads at different rotating frequencies are shown in Figure 11. From Figure 11, it can be seen that the power outputs of the piezoelectric element at the rotating frequencies of 0.79~14 Hz are in the range of 106~2308 μW, corresponding to a vehicle speed of 5.67~100.47 km/h with the assumed tire model. The output power is only from one piezoelectric element of one beam. If piezoelectric elements are mounted onto both sides of both beams, the total output power will nearly quadruple. As the reported power requirement for a wireless monitoring sensor is less than 20 μW [28], it shows that sufficient power can be obtained for the wireless monitoring sensor in the tire over a wide speed range. The power density of our designed harvester prototype is about 17.65 μW/cm^3^.

## 4. Conclusions and Discussion

A novel mechanism to transfer the rotational power into electrical power based on the piezoelectric cantilever beams and a hollow mass was presented in this paper. Based on the design, the harvester system can be mounted at a distance offset from the rotational center. A prototype was built to investigate the design. Based on the experimental results with the prototype, the power output of 106~2308 μW can be achieved for one piezoelectric cantilever beam at the rotating frequencies of 0.79~14 Hz, indicating that sufficient power can be harvested for a wireless monitoring sensor over a wide frequency range.

In our designed rotational energy harvester, the gap between the limiters and the hub of the harvester is an important parameter. At a higher rotation speed, the centrifugal force will be larger and the permitted gap distance will be smaller. Parametric studies on the gap distance, ring mass and dimensions of the piezoelectric beams will be explored in future works. Another issue for the rotational energy harvester is that a system at rest will not harvest any power. An approach to solve this issue is to utilize an energy storage device, which can accumulate the redundant energy from the power harvester when the system rotates at high frequency, and last but not least, the hollow mass used in the harvester can be something other than a ring-like mass. For example, it can be an ellipse-like mass; even more, it can be a non-closed profile, according to the requirements of the applications. However, to fulfill dynamic balance, the hollow mass should be symmetrically designed.

## Figures and Tables

**Table 1:** Parameters of the beam structure and piezoelectric elements.

Parameter	Value
*l_b_*, *w_b_*, *t_b_*	60, 50, 1.2 mm
*r*_*m*1_, *r*_*m*2_, *w_m_*	200, 170, 30 mm
*l_p_*, *w_p_*, *t_p_*	28.5, 50, 0.267 mm
*r_g_*	159 mm
*h_l_*	10 mm
*m_ring_*	2680 g
*C_p_*	113.5 nF
*x*_0_	1.5 mm
*r*_0_	1 mm
